# Clinical implementation of electron energy changes of Varian linear accelerators

**DOI:** 10.1120/jacmp.v10i4.2978

**Published:** 2009-10-27

**Authors:** Sean Zhang, Praimakorn Liengsawangwong, Patricia Lindsay, Karl Prado, Tzouh–Liang Sun, Roy Steadham, Xiaochun Wang, Mohammad R. Salehpour, Michael Gillin

**Affiliations:** ^1^ Department of Radiation Physics The University of Texas M.D. Anderson Cancer Center Houston Texas U.S.A.

**Keywords:** llinear accelerator, electron beam energy change

## Abstract

Modern dual photon energy linear accelerators often come with a few megavoltage electron beams. The megavoltage electron beam has limited range and relative sharp distal falloff in its depth dose curve compared to that of megavoltage photon beam. Its radiation dose is often delivered appositionally to cover the target volume to its distal 90% depth dose (d90), while avoiding the normal ‐ sometimes critical ‐ structure immediately distal to the target. Varian linear accelerators currently offer selected electron beams of 4, 6, 9, 12, 16 and 20 MeV electron beam energies. However, intermediate electron energy is often needed for optimal dose distribution. In this study we investigated electron beam characteristics and implemented two intermediate 7 and 11 MeV electron beams on Varian linear accelerators. Comprehensive tests and measurements indicated the new electron beams met all dosimetry parameter criteria and operational safety standards. Between the two new electron beams and the existing electron beams we were able to provide a choice of electron beams of 4, 6, 7, 9, 11, 12, 16 and 20 MeV electron energies, which had d90 depth between 1.5 cm and 6.0 cm (from 1.5 cm to 4.0 cm in 0.5 cm increments) to meet our clinical needs.

PACS number: 87.56.bd.

## I. INTRODUCTION

High energy electron beams generated from linear accelerators (linacs) have been used in radiation therapy for cancers since the 1970s. The basic physics and dosimetry of megavoltage electrons has been established for clinical use. The megavoltage electron beam has limited range and relative sharp distal falloff in its depth dose curve compared to that of megavoltage photon beams. The energy of the electron beam is often specified by its distal depth of 90 percent maximal dose (d90), where the radiation dose is often prescribed to cover the target volume.^(^ ^1^ ^–^ ^5^ ^)^

Linacs accelerate electrons linearly to high velocity and energy using high‐power microwaves. Low‐energy electrons are injected by an electron gun at one end of the accelerating waveguide, and accelerated along the guide to speeds approaching the speed of light. The electrons then enter a bending magnet assembly where they are redirected toward the beam's isocenter. In theory, only a 90° bend is needed to change the beam direction from horizontal to vertical. In practice, however, a 270° bending magnet is usually used, such as in Varian linacs. An energy filter or a slit is fitted in the magnet design to remove electrons that are not within 3% of the nominal peak of selected electron energy. By change the bending magnet's strength array, energy of the electron beam can be selected from an energy spectrum of accelerated electrons. The electrons will strike a scattering foil or a flattering filter on a flattering filter/scattering foil carrousel prior to exiting the linac.

We have six Varian 21EX linear accelerators in our facility. Each offers five electron energies. Three linear accelerators come with 6, 9, 12, 16, and 20 MeV electron energy beams, while the other three come with 4, 6, 9, 12, and 16 MeV electron energy beams. Together they provide six electron energy choices to meet our clinical needs. There are five electron scattering foil slots on the carrousel in Varian linac design. The 6 and 9 MeV electron beams share a slot and the other beam each has its own scattering foil. Each electron beam was turned and matched during its initial commissioning so that its d90 met our institution's specification.^(6)^ The d90 are 1.5, 2.0, 3.0, 4.0, 5.0, and 6.1 cm for the 4, 6, 9, 12, 16, and 20 MeV electron beams, respectively.

To have sufficient target coverage while avoiding critical normal structure immediately distal to the target volume, intermediate electron energy is often needed for optimal dose distribution. The tissue quivalent bolus placed on the patient's skin is used as a sub‐optimal alternative to change the depth of dose distribution. In this project we investigated and implemented two new electron energy beams for clinical use: E1 is between 6 and 9 MeV and its d90 is 2.5 cm; E2 is between 9 and 12 MeV and its d90 is 3.5 cm. These were labeled 7 MeV and 11 MeV, respectively. With these new additional electron beams we were able to provide a choice of electron beam with d90 depths of 1.5, 2.0, 2.5, 3.0, 3.5, 4.0, 5.0 and 6.1 cm (in 0.5 cm increments between 1.5 and 4.0 cm), to meet our clinical needs.

## II. MATERIALS AND METHODS

We first conducted a pilot study to investigate the characteristics of electron beam profiles of an intermediate electron energy beam, either by increasing the lower electron energy up or decreasing the higher electron energy down. The pilot study was divided into two parts: 1) to study the characteristics of the intermediate electron energy beam when the higher and lower electron energy beams (6 MeV and 9 MeV in this case) share the same scattering foil; 2) to study the intermediate electron energy beam characteristics when the higher and lower electron energy beams (9 MeV and 12 MeV in this case) use different scattering foils. Moreover, we also studied the effect on electron beam profile under the servo on and off conditions.

Based on the results from the pilot study, we cooperated with Varian to implement two new electron energy beams in our clinic. We first changed the bending magnet's current to alter the 6 MeV electron beam's d90 depth from 2.0 cm to 2.5 cm. We adjusted waveguide's RF power and electron beam current to maximize the electron beam output. We then steered and balanced the beam to optimize the beam profiles. The optimized beam parameters were saved to a new beam program card. We repeated the same procedures by tuning the 12 MeV electron beams down, changing its d90 depth from 4.0 cm to 3.5 cm. The linear accelerator that came with 6, 9, 12, 16 and 20 MeV electron beams had 7, 9, 11 16, and 20 MeV beams after electron beam energy change.

### A. Beam profile and depth dose measurements

We used the 3D Blue Phantom (Wellhofer, IBA Dosimetry America, Bartlett, TN, U.S.A.) with two CC04 cylindrical ion chambers placed in the radiation field. The relative reading of the scanning detector to that of the reference detector was recorded as beam profile. The IBA OmniPro‐Accept 6.2 software was used to position the chamber and the CU500E controller was connected to the connector box at the blue phantom tank and the OmniPro computer.

We measured the electron depth‐ionizing curve and the electron cross‐beam profile in water with source‐to‐surface of the water phantom distance at 100 cm (SSD technique). We used an electron cone size of 10×10cm for electron depth ionizing scanning and a cone size of 20×20cm for beam profiles. The AAPM TG‐51 protocol^(7)^ was applied to convert the electron ionization depth to the electron percentage depth‐dose. We shifted 1mm up from the CC04's central axis (half of the CC04 chamber radius) as the effective point of measurement.

AAPM TG‐25^(8)^ recommends determining the beam flatness and symmetry at the depth 95% dose beyond the depth of maximum dose. It is also recommended that the beam uniformity be evaluated near the surface and at the therapeutic range. In our institution the reference plane for beam uniformity measurements is set per Varian specifications, which are determined according to ICRU Report 21. The reference plane is set at a depth of 1.0 cm when the electron energy is below 10 MeV, and at a depth of 2.0 cm when the electron energy is 10 MeV or above. We also included scans at the distal 90% dose depth and 10×10cm cone size. For 6, 7 and 9 MeV, we scanned at depth of 1.0 cm and 2.5 cm (d90 of 7 MeV). For 9, 11 and 12 MeV, we scanned at a depth of 2.0 cm and 3.5 cm (d90 of 11 MeV).

The acceptance tolerance of beam flatness should not exceed ±5% (optimally within ±3%) over an area equal to or larger than 10×10cm, and the beam symmetry should be within 2% at any pair of points equal distant from the central axis, as recommended by AAPM TG‐25. Our institution's tolerance values are within ±3% and ±2% for the beam flatness and symmetry, respectively. The flatness is calculated by (1/2 mean) Variation over Mean (80%):

(1) $$\text{Flatness}=100*(D_{\text{max}}-D_{\text{min}})/(D_{\text{max}}+D_{\text{min}})$$

within flattened area (a) (defined below). $D_{\max}$ and $D_{\min}$ are defined as the maximum and minimum dose values compared with the dose at the central axis. Flattened areas (a) are defined as 80% of the field width.

Symmetry is expressed as maximum deviation between opposing halves of beams within the flattened area. It is calculated by point difference method, per Varian specifications. The point difference is defined as the difference between the doses delivered to any two points which are equidistant and symmetrical about the central axis and within the central 80% of the radial and transverse axes.

(2) Symmetry=100*Max(|Point L−Point R|)/Dcax

We smoothed the beam profiles with Bezier polynomials and applied the Varian protocol (as shown in equations above) to measure their flatness and symmetry. Moreover, we studied the difference of beam profile characteristics with the servo on and off conditions.

### B. Output factors

The $d_{\max}$ for each cutout and SSD as listed below is determined with depth‐dose scans. The output factors are then collected by placing the CC04 chamber at the specific $d_{\max}$ for that cutout and SSD in the water phantom (Wellhofer Blue Phantom). The charge readings are normalized to the charge reading of 10×10cm applicator, 100 cm SSD.

Output factors were measured for the following cone and cutout size in square for SSDs of 100, 105, 110, 115, and 120 cm as:

**Table:** 

Cone	Cutouts
6×6	2, 3, 4, 5, 6
10×10	2, 3, 4, 6, 8, 10
15×15	2, 3, 4, 6, 8, 10, 12, 15
20×20	2, 3, 4, 6, 8, 10, 12, 15, 20
25×25	2, 3, 4, 6, 8, 10, 12, 15, 20, 25

The output factors were linearly interpolated between the measured SSDs to generate tables from 100 to 120 cm in 1 cm increments. We measured all output factors for all cutouts and SSDs so that the virtual SSD is not used in our extended SSD MU calculations. The depth of $d_{\max}$ for each cutout and each SSD used in the output factor measurements are listed in Table 1.

**Table 1:** Depth of $d_{\max}$ for 7 MeV and 11 MeV beams at different SSDs for each cutout.

					*7 MeV*						
*Cutout size*	*2.0*	*3.0*	*4.0*	*5.0*	*6.0*	*8.0*	*10.0*	*12.0*	*15.0*	*20.0*	*25.0*
SSD 100	1.0	1.5	1.8	1.8	1.8	1.9	1.9	1.9	1.9	1.8	1.9
SSD 105	0.8	1.4	1.8	1.8	1.8	1.9	1.9	1.9	1.9	1.8	1.9
SSD 110	1.0	1.4	1.7	1.8	1.8	1.9	1.9	1.9	1.9	1.8	1.9
SSD 115	1.0	1.5	1.8	1.8	1.8	1.9	1.9	1.9	1.9	1.8	1.9
SSD 120	1.2	1.5	1.8	1.8	1.8	1.9	1.9	1.9	1.9	1.8	1.9
					*11 MeV*						
*Cutout size*	*2.0*	*3.0*	*4.0*	*5.0*	*6.0*	*8.0*	*10.0*	*12.0*	*15.0*	*20.0*	*25.0*
SSD 100	1.0	1.9	2.2	2.5	2.5	2.7	2.7	2.6	2.6	2.6	2.6
SSD 105	1.0	1.8	2.4	2.4	2.6	2.7	2.7	2.6	2.6	2.6	2.6
SSD 110	0.8	1.7	2.4	2.5	2.7	2.7	2.7	2.6	2.6	2.6	2.6
SSD 115	0.9	1.8	2.2	2.5	2.8	2.7	2.7	2.6	2.6	2.6	2.6
SSD 120	1.0	1.6	2.2	2.5	2.5	2.7	2.7	2.6	2.6	2.6	2.6

### C. Air gap factor

We measured the output factors of every cutout at 100, 105, 110, 115, and 120 cm SSD. We used the CC04 chamber at the $d_{\max}$ for each cutout measurement with the Blue Phantom. We calculated the air gap factor using the equations below:

(3) $$\begin{array}{l} \text{Output}\;(\text{FS},\;\text{SSD})=\text{output}\;({\text{FS}}_{\text{cal}},{\text{SSD}}_{\text{cal}})\times \text{output}\_\text{factor}\;({\text{FS}}_{\text{cal}},\;\text{FS})\times \\ {\left(({\text{SSD}}_{\text{cal}}+d_{\text{max}})/(\text{SSD}+d_{\text{max}})\right)}^{2}\times \times f_{\text{air}}(\text{FS},\;{\text{SSD}}_{\text{cal}},\;\text{SSD}) \end{array}$$

where $\text{FS}=\text{field size},\;{\text{FS}}_{\text{cal}}=\text{field size}$ at calibration (which is 10×10 cone), ${\text{SSD}}_{\text{cal}}=\text{SSD}$ at calibration (which is 100 cm), $f_{\text{air}}=\text{air gap factor}$.

Thus, the air gap factor, $f_{\text{air}}$, is calculated using this equation:

(4) $$f_{\text{air}}=\left(\frac{\text{rdg}(FS,\;\text{SSD})}{\text{rdg}(FS,\;100\;\text{cm}}\right){\left(\frac{\text{SSD}+d_{\text{max}}}{100\;\text{cm}+d_{\text{max}}}\right)}^{2}$$

### D. Beam profiles for treatment planning system commissioning

Beam data commissioning were performed according to the guidelines set by AAPM TG‐106.^(9)^ For each electron energy, we scanned at depths of 0.5R90, R90, R70, R50, Rp+2cm.

**Table:** 

Cone	Cutouts
6×6	2, 3, 4, 5, 6
10×10	8, 10
15×15	15
20×20	20
25×25	25

Cross‐plane profiles (in‐plane profiles assumed to be the same as cross‐plane profiles in planning system electron modeling) and percent‐depth doses were scanned for each of the above cone/cutout combinations. These profiles were smoothed (using Bezier filter for cross‐beam profiles, and least‐squares filter for PDD) and then imported into the Pinnacle Treatment Planning System (Pinnacle, ADAC, Milpitas, CA) for beam modeling.

**Figure 1:**
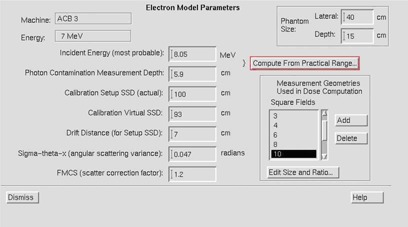
Parameters for Beam Modeling in Pinnacle.

The parameters for beam modeling in Pinnacle can be seen in the screen capture (Fig. 1). Of the above parameters, only incident energy and sigma‐theta‐x were different for 7 MeV vs. 11 MeV. Sigma‐theta‐x values were calculated from the in‐air profiles measured for 20×20 field size at 100, 105, 110, 115, and 120 cm SSD, as described in the Pinnacle documentation.

We also calculated the virtual SSD (from the FWHM of the in‐air profiles) to be 90.5 cm and 90.1 cm (for 7 MeV and 11 MeV, respectively). However, as had been done with previous commissioning of the standard electron energies, a default value (of 93 cm) was used in the Pinnacle modeling.

The calculated profiles were adjusted to match the measured profiles and input into the Pinnacle.

### E. diamond MU calculation program

The input data for Diamond (current clinical version) (K&S, Nashville, TN) MU calculation program was the output factor data measured and calculated as described above.

### F. Varian acceptance tests

Varian requested the following tests to de done. A Varian representative was present during the acceptance tests.

**Table:** 

Test #1:	Electron depth of ionization / depth dose.
Test #2:	Electron field fatness: The symmetry and fatness for electron energy E1 and E2, cone size 10×10,15×15,20×20,25×25, in‐plane and cross‐plane profiles.
Test #3:	Dosimetry.
	Short term reproducibility
	Reproducibility with MU settings
	Reproducibility with dose rate (Rep Rate)
	Reproducibility with gantry angle
	Dose rate accuracy
Test #4:	Electron applicator leakage per IEC 60601 $-2-1^{10}$.

## III. RESULTS & DISCUSSION

### A. With the same scattering foil

The distal depth of 90% maximum dose of the intermediate electron energy between 6 MeV and 9 MeV was roughly about 2.5 cm. To match with the 2.5 cm depth of the distal 90% dose, we increased the 6 MeV electron beam to 7.50 MeV. We decreased the 9 MeV electron beam to match its depth of distal 90% dose at 2.5 cm. Its final adjusted energy was 7.53 MeV. The fatness and symmetry parameters of the 7.5 MeV beam with cone size 20×20cm at the depth of 1.0 cm in both in‐plane and cross‐plane direction are shown in Table 2(a). These profiles were scanned with the servo at “on” position.

**Table 2(a):** The fatness and symmetry of both increasing the 6 MeV to 7.50 MeV and decreasing the 9 MeV to 7.53 MeV at SSD 100 cm and depth 1.0 cm with the servo at “on” position.

*Electron Energy*		*6 MeV to 7.50 MeV*	*9 MeV to 7.53 MeV*
In‐plane	Symmetry	0.50%	0.70%
	Flatness	0.70%	0.90%
Cross‐plane	Symmetry	0.50%	0.80%
	Flatness	0.90%	0.90%

**Table 2(b):** The fatness and symmetry of both increasing the 9 MeV to 10.44 MeV and decreasing the 12 MeV to 10.54 MeV at SSD 100 cm and depth 2.0 cm with the servo at “on” position.

*Electron Energy*		*9 MeV to 10.44 MeV*	*12 MeV to 10.54 MeV*
In‐plane	Symmetry	0.40%	0.40%
	Flatness	**3.10%**	0.90%
Cross‐plane	Symmetry		0.90% 0.60%
	Flatness	2.40%	1.60%

As shown in Table 2(a), the characteristics of electron beams with the same scattering foil have small differences. Their parameters of fatness and symmetry of beam profiles for either increasing the 6 MeV to 7.50 MeV or decreasing the 9 MeV to 7.53 MeV were within the acceptance tolerance, (±3% for fatness and ±2% for symmetry (standard set at our facility). (TG40 recommends ±3% for fatness and symmetry.)

### B. With the different scattering foil

The distal depth of 90% maximum dose of the intermediate electron energy between 9 MeV and 12 MeV was roughly about 3.5 cm. In order to match with the 3.5 cm depth of the distal 90% dose, we increased the 9 MeV to 10.44 MeV. We also decreased the 12 MeV until its distal 90% dose depth matched with 3.5 cm. Its final adjusted energy was 10.54 MeV. The fatness and symmetry parameters of 10.5 MeV beams with cone size 20×20cm at the depth 2.0 cm in both in‐plane and cross‐plane directions are shown in Table 2(b). These profiles were scanned with the servo at “on” position.

In this case, it was observed that increasing the 9 MeV beam to 10.44 MeV beam would give the worse results with respect to the fatness and symmetry of beam profiles compared to decreasing the 12 MeV beam to 10.54 MeV beam. The fatness of beam profile in the in‐plane direction had higher value than the acceptance tolerance value (3.10%>3.00%). The details of the parameter values are in Table 2(b).

The results of the pilot study demonstrated that the profiles of the electron beam E1 have little difference between tuning 6 MeV up and 9 MeV down. However the electron beam E2 has better fatness profiles when tuning 12 MeV beam down than those with 9 MeV beam being tuning up. The electron scattering foil design in a modern linac was optimized to produce the most uniform electron fluence across the treatment field. In the Varian linac design, the scattering foil to spread the 6 and 9 MeV electron beams was optimized for the beam energy in between, as two electron beams share the same scattering foil. So tuning the beam energy up from 6 MeV or tuning it down from 9 MeV resulted in little difference in beam fatness and symmetry. However, by tuning the beam up from 9 MeV and tuning the beam down from 12 MeV, we found the 12 MeV scattering foil optimized for 12 MeV beam was better suited for the 11 MeV beam than the 6 and 9 MeV scattering foil designed for the 7 MeV beam. Since electrons scatter inversely proportional to their energy, the scattering foil designed for 6 and 9 MeV beams needs less scattering power than that designed for the 12 MeV beam. Using the lower energy foil for high‐energy electrons will create round shoulders in its beam profiles.

### C. Central‐axis depth dose of 7 MeV and 11 MeV.

The characteristics of 7 MeV and 11 MeV electron beams for a 10×10 applicator size are shown in Table 3. Definitions of some of these values are as follows: $R_{50}$ is taken as the depth of 50% of maximum dose along the central axis; $E_{p,0}=0.22+1.98R_{p}+0.0025R_{p}^{2};\;<E{>}_{0}=2.33R_{50}$; the surface dose $D_{s}$ was evaluated at 0.02 cm depth; the bremsstrahlung dose $D_{x}$ was determined using the deepest point measured within the scan.

**Figure 2:**
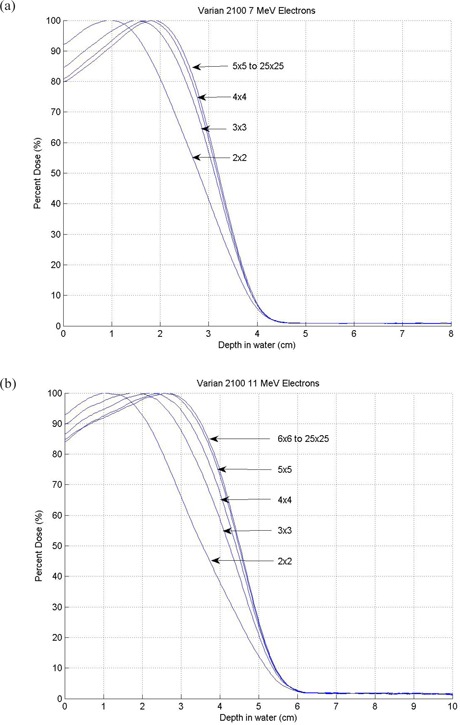
New Electron Beams Percent Depth Dose: 2(a). 7 MeV electron beam percent depth dose; 2 (b) 11 MeV electron beam percent depth dose.

The details of the central axis depth dose curves for all cutout inserts and applicator sizes are shown in Fig. 2(a) for 7 MeV and in Fig. 2(b) for 11 MeV.

The mean energy of electron beam at the surface of the phantom was 7.51 MeV for 7 MeV electron beam and 10.44 MeV for 11 MeV electron beam, as we had set out to achieve. The distal d90 depths were 2.5 cm and 3.5 cm for 7 MeV and 11 MeV beams, respectively. With these two new electron beams we were able to provide a choice of electron beam which had d90 depths of 1.5, 2.0, 2.5, 3.0, 3.5, 4.0, 5.0 and 6.1 cm, in 0.5 cm increments between 1.5 and 4.0 cm, to meet our clinical needs.

In our breast services, we use electron beam to treat internal mammary chain (IMC) nodes for advanced‐staged breast cancer patients. We use an appositional electron beam to match ipsilateral tangent photo beams. As the heart is often immediately below the IMC nodes, it is critical to have an electron beam with its d90 depth dose just covering the IMC nodes, while avoiding to treat the heart below. With 0.5 cm increments of d90 depth dose, rather than 1.0 cm increments as with existing electron beam choices, we will be able to have better dose distribution for IMC nodes treatment.

**Table 3:** Electron beam characteristics of the 7 MeV and 11 MeV beams for a 10×10 applicator size.

*Nominal Energy (MeV)*	*7*	*11*
$E_{p,0}(\text{MeV})$	8.09	11.06
${<E>}_{0}(\text{MeV})$	7.51	10.44
Surface dose $D_{s}\;(\%)$	78.5	83.6
Proximal $R_{90}(\text{cm})$	0.94	0.85
$R_{100}(\text{cm})$	1.88	2.69
Distal $R_{90}(\text{cm})$	2.49	3.48
$R_{80}(\text{cm})$	2.73	3.83
$R_{50}(\text{cm})$	3.22	4.48
$R_{20}(\text{cm})$	3.71	5.09
$R_{10}(\text{cm})$	3.93	5.40
$R_{p}\;(\text{cm})$	3.96	5.44
$D_{x}(\%)$ (last‐point method)	0.70	1.30

The electron beam percentage depth dose increases with its field size until it reaches its critical field size. All beams with critical field size or larger have the same depth dose curve. For the 7 MeV electron beam, the critical field size is 5×5cm, and 6×6cm for the 11 MeV beam (see Fig. 2).

### D. output factors and air gap factors

**Figure 3:**
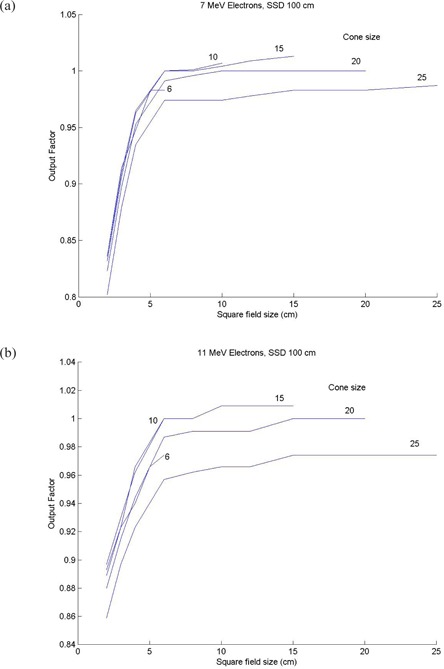
New Electron Beams Output Factors: 3(a) 7 MeV electron output factors; 3(b) 11 MeV electron output factors.

Data for the output factor for 7 and 11 MeV as a function of field size for different cone sizes are provided in Fig. 3(a) and Fig. 3(b). The output factors generally increase dramatically with field size until its critical field size, and then leveled off. The critical field sizes are 5 and 6 cm for the 7 MeV and 11 MeV beams, respectively. The output factors decrease with cone size with all cones larger than 10 cm, as shown. The air gap factors were calculated using the output factors measured at 100, 105, 110, 115, and 120 cm SSD, as shown in Table 4.

**Table 4:** 7 MeV and 11 MeV electron beam air gap factors.

			*7 MeV*					
*Field Size (cm^2^)*	*2*	*3*	*4*	*6*	*10*	*15*	*20*	*25*
SSD (cm)								
100	1.000	1.000	1.000	1.000	1.000	1.000	1.000	1.000
105	0.797	0.862	0.883	0.897	0.909	0.900	0.902	0.893
110	0.583	0.721	0.766	0.797	0.808	0.810	0.814	0.811
115	0.415	0.579	0.647	0.706	0.732	0.727	0.733	0.733
120	0.311	0.471	0.555	0.635	0.668	0.667	0.673	0.671
				*11 MeV*				
*Field Size (cm^2^)*	*2*	*3*	*4*	*6*	*10*	*15*	*20*	*25*
SSD (cm)								
100	1.000	1.000	1.000	1.000	1.000	1.000	1.000	1.000
105	0.946	0.969	0.968	0.982	0.985	0.990	0.992	0.988
110	0.840	0.935	0.942	0.967	0.977	0.981	0.986	0.984
115	0.703	0.869	0.906	0.944	0.968	0.965	0.973	0.972
120	0.604	0.815	0.876	0.934	0.958	0.959	0.969	0.966

### E. Varian tests and safety and operational standards

Since 7 MeV and 11 MeV electron beams were first implemented in Varian linear accelerators, we have cooperated with Varian and performed a set of comprehensive tests.^(10)^ The tests included all Varian's electron beam acceptance tests and our institutional electron beam commissioning tests. We also performed electron beam leakage tests per IEC Standard,^(10)^ which was typically done for every Varian's electron energy at Varian's test site. All beam parameter and dosimetry interlocks were tested for new electron beam's program board. After all tests were satisfied, the Varian engineer changed the electron beam label 6 MeV to 7 MeV and 12 MeV to 11 MeV at our two linacs. To implement the new electron beam energies, we also changed MosaiQ (our recording and verifying system) database, input the beam data to the Pinnacle (the treatment planning system) and to the Diamond (our independent electron Monitor Unit calculation system). After a successful dry run of all components from the treatment simulation to the plan for delivery, we put the new electron energy beam into the clinic two years after the start of the project.

## IV. CONCLUSIONS

We have successfully implemented two new electron beams in Varian linear accelerators. The distal depths of 90% maximum dose of the beams were 2.5 cm and 3.5 cm. Comprehensive tests and measurements demonstrated that the beams met all electron beam dosimetry criteria and operational safety standards. With these new electron beams we were able to provide a selection of electron beams with the distal depths of 90% maximum dose from 1.5 cm to 4.0 cm in 0.5 cm increments, to optimize electron beam treatment dosimetry.
